# Adult microbiota‐deficient mice have distinct dendritic morphological changes: differential effects in the amygdala and hippocampus

**DOI:** 10.1111/ejn.13291

**Published:** 2016-07-08

**Authors:** Pauline Luczynski, Seán O. Whelan, Colette O'Sullivan, Gerard Clarke, Fergus Shanahan, Timothy G. Dinan, John F. Cryan

**Affiliations:** ^1^ APC Microbiome InstituteUniversity College CorkRoom 3.86, Western Gateway Building, CorkIreland; ^2^ Department of Anatomy and NeuroscienceUniversity College CorkCorkIreland; ^3^ Department of Psychiatry and Neurobehavioural ScienceUniversity College CorkCorkIreland

**Keywords:** brain volume, dendritic spines, design‐based stereology, germ‐free mouse, microbiota–gut–brain axis

## Abstract

Increasing evidence implicates the microbiota in the regulation of brain and behaviour. Germ‐free mice (GF; microbiota deficient from birth) exhibit altered stress hormone signalling and anxiety‐like behaviours as well as deficits in social cognition. Although the mechanisms underlying the ability of the gut microbiota to influence stress responsivity and behaviour remain unknown, many lines of evidence point to the amygdala and hippocampus as likely targets. Thus, the aim of this study was to determine if the volume and dendritic morphology of the amygdala and hippocampus differ in GF versus conventionally colonized (CC) mice. Volumetric estimates revealed significant amygdalar and hippocampal expansion in GF compared to CC mice. We also studied the effect of GF status on the level of single neurons in the basolateral amygdala (BLA) and ventral hippocampus. In the BLA, the aspiny interneurons and pyramidal neurons of GF mice exhibited dendritic hypertrophy. The BLA pyramidal neurons of GF mice had more thin, stubby and mushroom spines. In contrast, the ventral hippocampal pyramidal neurons of GF mice were shorter, less branched and had less stubby and mushroom spines. When compared to controls, dentate granule cells of GF mice were less branched but did not differ in spine density. These findings suggest that the microbiota is required for the normal gross morphology and ultrastructure of the amygdala and hippocampus and that this neural remodelling may contribute to the maladaptive stress responsivity and behavioural profile observed in GF mice.

## Introduction

A rapidly growing body of evidence points to a role of the microbiota in the regulation of brain and behaviour. The microbiota–gut–brain axis represents a bidirectional network of communication between the intestinal microbiota and the brain (Rhee *et al*., 2009; Bercik *et al*., 2012; Cryan & Dinan, 2012; Dinan & Cryan, 2012; Sampson & Mazmanian, 2015). The gut microbiota is required for development of the hypothalamic–pituitary–adrenal (HPA) axis, optimal stress responsivity and social cognition (Neufeld *et al*., 2011; Clarke *et al*., 2013; Desbonnet *et al*., 2014). Dysregulation of the microbiota–gut–brain axis may contribute to the development of psychiatric and gastrointestinal diseases, a link supported by the comorbidity found between anxiety disorders and irritable bowel syndrome (Fond *et al*., 2014) as well as the abnormal composition of gut microbiota in patients with autism (Mayer *et al*., 2014).

Germ‐free mice (GF; microbiota‐deficient from birth) have provided critical insights into the role of the microbiota in regulating brain function (Luczynski *et al*., 2016). We and others have previously shown that GF mice exhibit exaggerated HPA axis responses to acute stressors, reduced anxiety‐like behaviours and deficits in social cognition (Sudo *et al*., 2004; Heijtz *et al*., 2011; Neufeld *et al*., 2011; Clarke *et al*., 2013; Desbonnet *et al*., 2014; Arentsen *et al*., 2015), effects which are influenced by the amygdala and hippocampus (Sah *et al*., 2003; Ledoux, 2007; Tovote *et al*., 2015). Indeed, signalling between the basolateral amygdala (BLA) and the ventral hippocampus modulates both anxiety and social behaviours (Felix‐Ortiz *et al*., 2013; Allsop *et al*., 2014; Felix‐Ortiz & Tye, 2014).

Studies have documented altered amygdalar and hippocampal structure in both humans and animals with exaggerated stress responses. Changes in amygdalar and hippocampal volume have been associated with anxiety disorders in humans and with early‐life stress in rodents (De Bellis *et al*., 2000; Isgor *et al*., 2004; Salm *et al*., 2004; Salzer & Weniger, 2010; Machado‐de‐Sousa *et al*., 2014). There is also evidence showing long‐lasting dendritic hypertrophy of excitatory neurons in the BLA of rodents repeatedly exposed to stressors (Vyas *et al*., 2002, 2004, 2006). In contrast, repeated stress induces dendritic atrophy in hippocampal neurons (Magariños & McEwen, 1995a; Vyas *et al*., 2002). Thus, although the mechanisms underpinning the ability of the gut microbiota to influence stress responsivity and behaviour remain to be elucidated, many lines of evidence point to the amygdala and hippocampus as likely targets.

This study is based on the hypothesis that the GF phenotype could be due to morphological changes in amygdalar and hippocampal subregions with implications for neural output. To this end we determined if the presence of a gut microbiota is necessary for normal structure of the amygdala and hippocampus. We firstly utilized design‐based stereology to compare the volumes of well‐defined brain regions between adult GF and conventionally colonized (CC) animals. Secondly, we evaluated the dendritic morphology of single neurons in the BLA and hippocampus, regions known to undergo neuronal remodelling and to regulate both anxiety and social behaviours (Vyas *et al*., 2002; Allsop *et al*., 2014).

## Materials and methods

### Animals

GF and CC Swiss Webster breeding pairs were supplied by Taconic (Germantown, New York, USA) and first‐generation male offspring were used in all experiments. GF mice were housed 3–5 per cage in flexible film gnotobiotic isolators held under controlled conditions (temperature 21 ± 1 °C, 55–60% relative humidity) and under a 12 h light/dark cycle. CC mice were housed 2–5 per cage in the standard animal facility under the same controlled conditions and light/dark cycle as the GF animals. Only one pair of CC animals was housed 2 per cage. Both GF and CC mice received the same autoclaved pelleted diet (Special Diets Services, product code 801010). Cohorts of GF and CC mice were randomly allocated to two groups: one for stereology and one for dendritic morphology. Animals were killed at 9–10 weeks of age. A total of 26 mice were used in this study. All experiments were performed in accordance with the guidelines of European Directive 86/609/EEC and the Recommendation 2007/526/65/EC and were approved by the Animal Experimentation Ethics Committee of University College Cork.

### Stereological volume estimation

Amygdalar, hippocampal and total brain volume were estimated in both hemispheres using Cavalieri's principle (Gundersen *et al*., 1988). Brain structures were identified using the Paxinos & Franklin (2001) atlas as a guide. For all analyses, preliminary studies were carried out to establish inter‐rater reliability with a disagreement of less than 10% on each brain structure volume.

#### Histological preparation

GF and CC animals were terminally anesthetized with sodium pentobarbital and perfused transcardially with 0.1 m phosphate buffer (pH 7.4), followed by 4% paraformaldehyde in 0.1 m phosphate buffer. After perfusion, the brains were carefully removed and postfixed for 24 h in a 4% paraformaldehyde 0.1 m phosphate buffer solution after which the brains were cryoprotected in a 30% sucrose solution for 48 h and flash frozen in isopentane. The brains were stored at −80 °C before being sliced. Brains were sectioned coronally at 40 μm on a cryostat and stained with thionin. Slides were then coded to obscure the experimental group of each animal until the final step of statistical analysis.

#### Amygdalar volume

**Figure 1:**
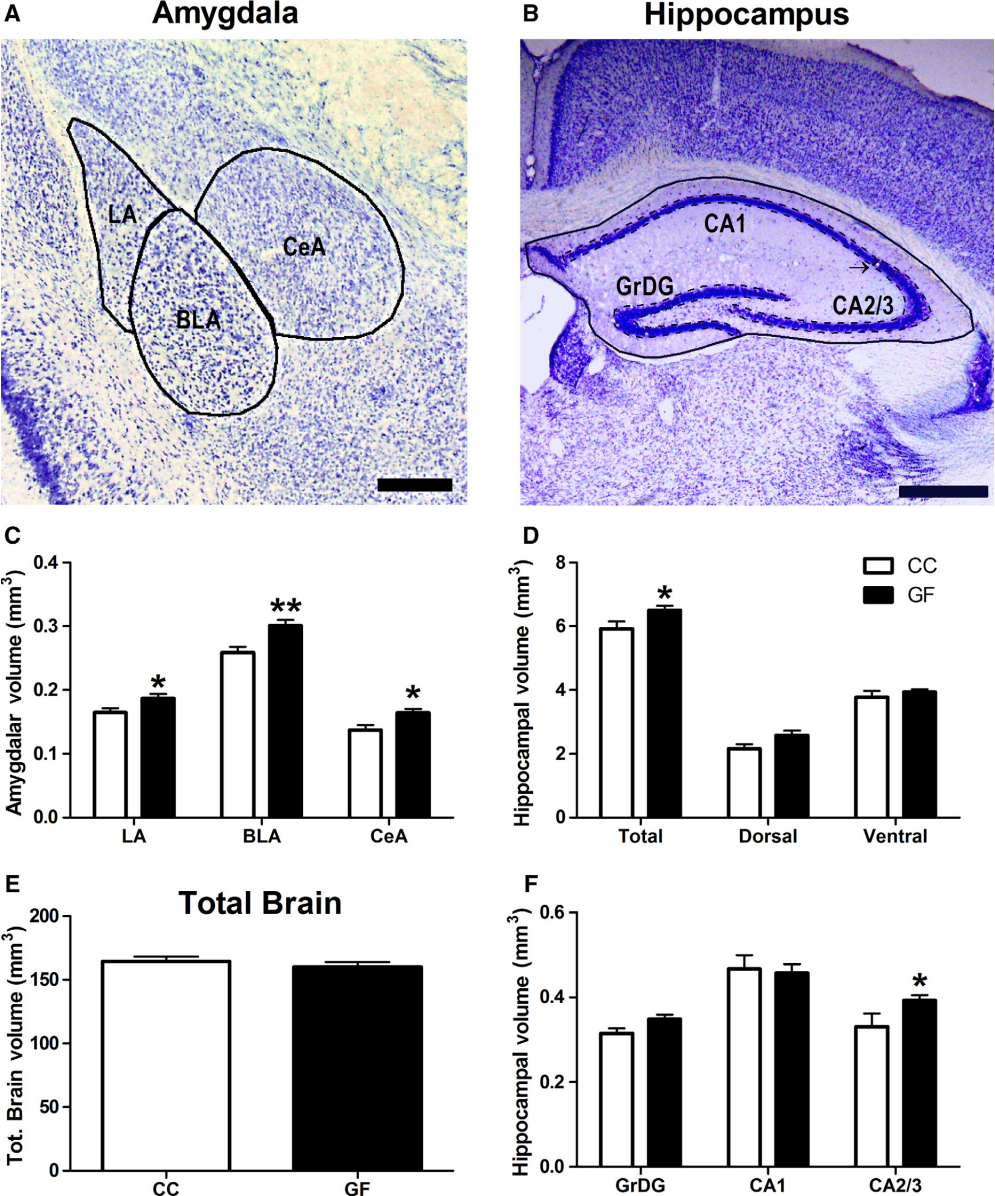
Amygdalar and hippocampal volumetric expansion in GF mice. (A) Representative photomicrograph of thionin‐stained section of the amygdala (A; scale bar = 0.25 mm) and the dorsal hippocampus (B; scale bar = 0.5 mm). The volumes of the defined (black lines) subregions of interest were estimated using Cavalieri's principle. The arrow indicates the border between CA1 and CA2/3. (C) The LA, BLA and CeA were significantly enlarged in GF compared to CC mice. (D, F) When compared to controls, the total volume of the hippocampus was larger in GF mice. (E) There was no difference in total brain volume between CC and GF mice. CC,* n* = 5–6; GF,* n* = 5–8. *P ≤* *0.05, **P <* *0.01.

The volumes of the lateral (LA; LAVL + LAVM + LADL), basolateral (BLA; BLA + BLP) and central nucleus of the amygdala (CeA; CeL + CeM) were estimated. The first rostrocaudal appearance of the BLA (Bregma −0.59 mm) was chosen as the sample starting point and the termination of the LA was chosen as the end point (Bregma −2.45 mm). For each amygdala, 7 to 12 evenly spaced serial sections with a randomized start were studied (periodicity of every third or fourth slice; section increment of 0.12 or 0.16 mm). The region of interest was digitally outlined in each section (4× magnification; N.A. 0.1; Fig. 1A) and converted into area values (mm^2^) using Fiji Is Just ImageJ (FIJI) software (Schindelin *et al*., 2012). The area values were then combined with the cut section thickness (0.04 mm) and section increment to compute the volumes of the sampled amygdala nuclei. In three GF animals, only one hemisphere was evaluated due to damaged amygdalar tissue.

#### Hippocampal volume

The volumes of the dorsal and ventral hippocampal formation, the granular layer of the dentate gyrus (GrDG) as well as the hippocampal fields were estimated. The hippocampal formation included the hippocampus proper (Ammon's horn), the dentate gyrus and the subicular complex. The first rostrocaudal appearance of CA3 (Bregma −0.94 mm) was chosen as the sample starting point and the termination of the GrDG (Bregma −4.03 mm) was chosen as the end point. The transition point between the dorsal and ventral hippocampus was taken to be the first full ventral extension of CA3 (Bregma −2.70 mm). The GrDG and the hippocampal field volumes were estimated within the rostrocaudal coordinates containing the total hippocampal formation. The hippocampal fields included the stratum oriens, stratum pyramidale and stratum radiatum. CA2 and CA3 were grouped together (CA2/3) as their anatomical borders are not easily distinguishable using nissl staining. For each hippocampal structure, 17–25 evenly spaced sections with a randomized start were studied (periodicity of every third slice, section increment of 0.12 mm). The regions of interest were outlined (4× magnification; N.A. 0.10; Fig. 1B) and volume was calculated as described above. In one CC animal, only one hippocampus was evaluated due to damaged tissue.

#### Total brain volume

The volume of the fore‐ and midbrain (the whole brain excluding the olfactory bulb and cerebellum; approx. Bregma 2.80–4.84 mm) was estimated as described above. Briefly, for each brain, 13–15 evenly spaced serial sections with a randomized start were studied (periodicity of every 10th slice; section increment of 0.4 mm). The perimeter of each hemisphere was digitally outlined in each section (2× magnification, N.A. 0.08) and the total brain volume was calculated. Two GF animals were excluded from the brain volume analysis because tissue damage was extensive and prevented the accurate measurement of the perimeter of one hemisphere.

### Dendritic morphology and spine density

#### Golgi‐Cox stain

GF and CC animals were terminally anesthetized with sodium pentobarbital and perfused transcardially with a vascular rinse (0.9% saline solution). Immediately following perfusion, brains were removed from the skull and Golgi‐Cox staining was performed with a whole‐brain staining kit (Bioenno Tech, Irvine, CA, USA) using a 10‐day impregnation period. Using a vibratome, 150 μm coronal sections were collected serially and mounted on gelatin subbed slides. To visualize amygdala cytoarchitecture, slices were lightly stained for Nissl bodies with thionin. Slices were then dehydrated (alcohol and xylene) and coverslipped with DPX. Slides were coded to obscure the experimental group of each animal until the final step of statistical analysis.

#### Classification of neuronal subtypes

Spiny pyramidal neurons and aspiny interneurons in the BLA were selected for analysis on the basis of morphological criteria described in the literature (McDonald, 1982; Sah *et al*., 2003; Klenowski *et al*., 2015). Approximately 80% of the neurons in the BLA are excitatory projection cells, and can be accurately subclassified as pyramidal or stellate based on distinct morphological and electrophysiological characteristics alone (McDonald, 1982, 1992; Millhouse & DeOlmos, 1983; Washburn & Moises, 1992; Rainnie *et al*., 1993; Faber *et al*., 2001; Sah *et al*., 2003). BLA pyramidal neurons (also termed class I, principal or pyramidal‐like) have a large triangle shaped soma and three to seven spiny dendrites emanating from the soma, with one or two of these dendrites being more prominent and similar to the apical dendrite of cortical neurons (McDonald, 1982; Sah *et al*., 2003; Klenowski *et al*., 2015). However, unlike the pyramidal neurons in the cortex or hippocampus, these cells are not oriented in one plane, the apical and basilar dendrites are of equivalent lengths, and the distal dendrites do not have a terminal elaboration but taper rapidly. Only BLA pyramidal neurons with a single primary apical dendrite were chosen for analysis. The second group of cells found within the BLA have a smaller cell body, two to six primary dendrites lacking spines, and a spherical dendritic arbour (McDonald, 1982; Millhouse & DeOlmos, 1983; Sah *et al*., 2003; Klenowski *et al*., 2015). These neurons (here termed aspiny interneurons but also known as aspiny stellate, or class II) are GABAergic local circuit interneurons which express calcium‐binding proteins and an inhibitory electrophysiological response profile (McDonald, 1992; Washburn & Moises, 1992; Rainnie *et al*., 1993; Lang & Paré, 1998; Kemppainen & Pitkänen, 2000; McDonald & Betette, 2001; Sah *et al*., 2003). We used the presence of an apical dendrite, soma diameter and presence of spines as a well‐validated means to distinguish pyramidal dendrites from aspiny interneurons. This method of morphological classification between BLA neuron subtypes has been widely utilized in the literature (McDonald, 1982; Millhouse & DeOlmos, 1983; Vyas *et al*., 2002, 2006; Rubinow *et al*., 2009; Qin *et al*., 2011; Bringas *et al*., 2013; Klenowski *et al*., 2015).

In the ventral hippocampus, CA3 pyramidal neurons were selected for analysis based on their distinct morphological features. Hippocampal pyramidal neurons have distinct apical and basilar dendritic arbours and a triangular‐shaped cell body (Spruston, 2008). Both short‐ and long‐shaft pyramidal neurons were analysed (Fitch *et al*., 1989).

In the dorsal dentate gyrus of the hippocampus, granule cells were identified by their cone‐shaped dendritic tree and lack of basilar dendrites. They were differentiated from basket cells by their elliptical, markedly smaller cell body (Amaral *et al*., 2007). Granule cells were selected from the outer two thirds of the granule cell layer to minimize the potential for analysing an immature neuron.

#### Analysis of dendritic arbourization

The analysis of BLA neurons was restricted to those located between Bregma −0.71 mm and −2.45 mm. The analysis of ventral hippocampal neurons was restricted to those located in CA3 between Bregma −2.70 mm and −3.52 mm. The analysis of dentate granule cells was restricted to those located in the dorsal hippocampus, between Bregma −0.94 mm and −2.70 mm. Both apical and basilar dendrites were characterized for pyramidal neurons. To be included in the analysis, the Golgi‐impregnated neurons had to satisfy the following criteria as described by Vyas *et al*. (2002): (i) absence of truncated dendrites, (ii) dark and uniform staining along all dendritic projections and (iii) relatively isolated from neighbouring stained neurons to avoid interference with dendritic reconstruction.

Three pyramidal and aspiny inhibitory neurons were reconstructed in each hemisphere of each animal, with the exception of one CC animal for which all six BLA pyramidal neurons were from the right hemisphere due to tissue damage to the opposite BLA. For the same reason, all six hippocampal pyramidal neurons from one CC and one GF animal were located in the right hemisphere. Two granule cells, one from the infrapyramidal blade and one from the suprapyramidal blade, were reconstructed in each hemisphere of each animal. The neurons were viewed using an Olympus AX70 Provis upright bright‐field microscope with an Olympus DP50 camera (Mason, Ireland). Images of each dendritic arbour were taken at 40× magnification (N.A. 1.0) with an oil immersion objective at 1 or 2 μm intervals throughout the entire dendritic arbour. Colour‐inverted images were then stacked and reconstructed manually in 3D using the Neurofilament tool in Imaris (Bitplane, Switzerland). Total dendritic length and branching were calculated by the software automatically. Sholl analysis, the measurement of the extent and complexity of dendritic material as a function of radial distance from the soma, was performed on 2D images of the traced dendritic arbours using the plug‐in for FIJI (Schindelin *et al*., 2012; Ferreira *et al*., 2014). The radius step size was 10 μm for aspiny interneurons and dentate granule cells and 20 μm for pyramidal neurons.

#### Analysis of spine density

Spine density was measured in all BLA and hippocampal pyramidal and granule neurons whose dendritic trees were reconstructed to quantify dendritic arbourization. This analysis was not performed on BLA interneurons as they have few to no spines (McDonald, 1982; Millhouse & DeOlmos, 1983; Sah *et al*., 2003; Klenowski *et al*., 2015). Dendritic spines were counted manually from image stacks taken at a magnification of 100× (N.A. 1.4). A dendritic segment of approximately 20 μm (branch order ≥ 2) was selected that possessed consistent and dark impregnation along the entire extent of the dendrite. All protrusions, regardless of their morphological characteristics, were counted as spines if they were continuous with the dendritic shaft. Two to three apical and basilar dendritic segments from each BLA and hippocampal pyramidal neuron were analysed. Three dendritic segments from each dentate granule cell were analysed. Spines were classified using RECONSTRUCT image analysis software developed by Risher *et al*. (2014). Briefly, each individual spine's length and width were measured and, based on these measurements, spines were classified in the following subtypes: filopodia (length value > 2 μm), mushroom (width value > 0.6 μm), thin (length‐to‐width ratio > 1) or stubby (length‐to‐width ratio ≤ 1). All values were calculated by averaging the spine densities of the sampled segments for each neuron.

### Statistical analysis

All data are expressed as means + or ± 1 SEM. Animal means were used for all analyses of dendritic morphology and spine density data. The unpaired Student's *t*‐test (α = 0.05) was used to assess the reliability of group differences (CC vs. GF; right vs. left hemisphere) in volume, total dendritic length, number of branches and spine morphology and density. For Sholl analyses, group differences were tested for significance with a two‐way mixed‐design anova, with radial distance from the soma as a within‐group factor. *Post hoc* comparisons were made using a Bonferroni's correction, with a significance set at *P *<* *0.05. Percentage changes were calculated with respect to corresponding control values.

## Results

### Stereological volume estimation

Data were obtained from mice with at least one intact and undamaged amygdala or hippocampus (CC: *n* = 5–6; GF: *n* = 5–8). There were no significant hemispheric differences in amygdalar or hippocampal volume in either CC or GF mice (*P *>* *0.05 for all regions), therefore, hemisphere means were used for these analyses.

#### Amygdalar volume

When compared to CC mice, the amygdala of GF animals showed significant volumetric expansion of all three subregions studied (Fig. 1C). In these animals, the mean volume was increased by 13% in the LA (*t*_12_ = 2.21, *P* = 0.047), 16% in the BLA (*t*_12_ = 3.13, *P* = 0.0087) and 20% in the CeA (*t*_12_ = 2.74, *P* = 0.018). Importantly, there was no significant difference in total brain volume between groups (*t*_10_ = 0.75, *P* = 0.47; Fig. 1E).

#### Hippocampal volume

The total hippocampus was enlarged by 10% in GF mice; however, there was no significant group difference in the dorsal or ventral hippocampal volume (total: *t*_9_ = 2.26, *P* = 0.051; dorsal: *t*_9_ = 2.02, *P* = 0.074; ventral: *t*_9_ = 0.76, *P* = 0.47; Fig. 1D). GF and CC mice did not significantly differ in the volumes of the GrDG and CA1 hippocampal field (GrDG: *t*_9_ = 1.85, *P* = 0.097; CA1: *t*_9_ = 0.56, *P* = 0.59; Fig. 1F). The CA2/3 hippocampal field was 19% larger in GF compared to CC mice (CA2/3: *t*_9_ = 2.55, *P* = 0.031; Fig. 1F).

### Dendritic morphology and spine density

Morphometric analyses were performed on the entire dendritic arbour of Golgi‐stained aspiny interneurons and pyramidal neurons with cell bodies in the BLA and the ventral CA3 of the hippocampus. For each animal, 4–6 neurons of each type were analysed for dendritic length and branching (CC: 48 BLA interneurons, 36 BLA pyramidal neurons, 30 hippocampal pyramidal neurons, 24 dorsal dentate granule cells; GF: 48 BLA interneurons, 42 BLA pyramidal neurons, 36 hippocampal pyramidal neurons, 24 dorsal dentate granule cells).

#### Basolateral amygdalar aspiny interneuron dendritic morphology

**Figure 2:**
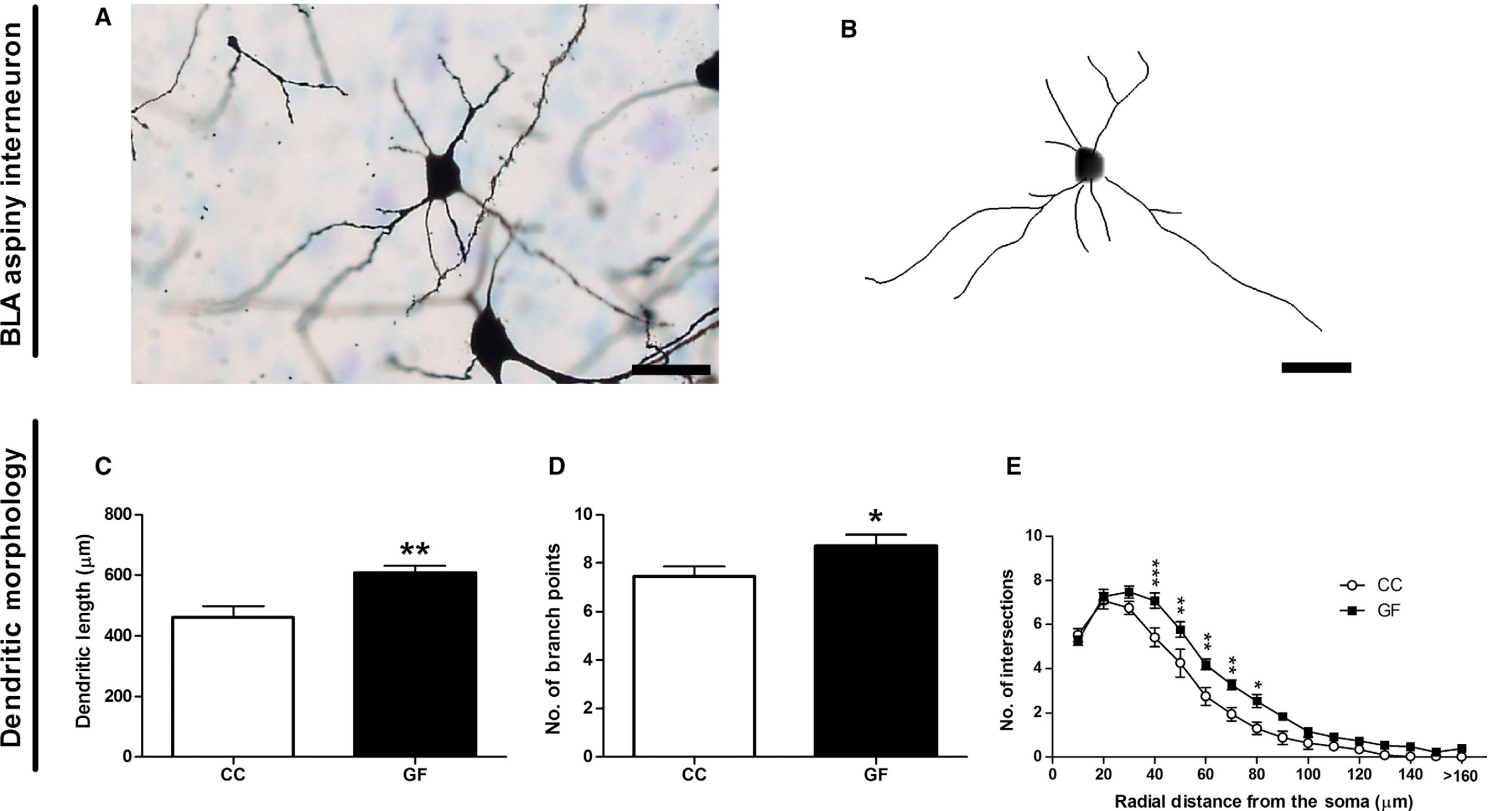
Dendritic hypertrophy of BLA aspiny interneurons in GF mice. (A, B) Representative photomicrograph of a Golgi‐stained aspiny interneuron in the BLA (A) and resulting computer‐assisted morphometric reconstruction (B). Scale bars = 25 μm. (C, D) In GF mice, aspiny interneurons were significantly longer (C) and had an increased number of branch points (D). (E**)** Sholl analysis revealed an elongation of intermediate dendrites (40–80 μm from the soma) in GF mice. *n* = 8 for both groups. *P ≤* *0.05, **P <* *0.01, ***P <* *0.001.

BLA aspiny interneurons showed significant dendritic hypertrophy in GF compared to CC mice (Fig. 2). GF status increased the total dendritic length by 32% (*t*_14_ = 3.31, *P* = 0.0051; Fig. 2C) and the total number of branch points by 17% (*t*_14_ = 2.11, *P* = 0.053; Fig. 2D). Two‐dimension Sholl analysis revealed a significant interaction of group (CC vs. GF) and distance from the soma on aspiny interneuron dendritic density (*F*_15, 210_ = 2.96, *P *<* *0.001); *post hoc* analyses revealed that the greatest dendritic extension (31–97%; *P *<* *0.05) occurred within the intermediate portions (40–80 μm) of the arbour (Fig. 2E).

#### Basolateral amygdalar pyramidal neuron dendritic morphology and spine density

**Figure 3:**
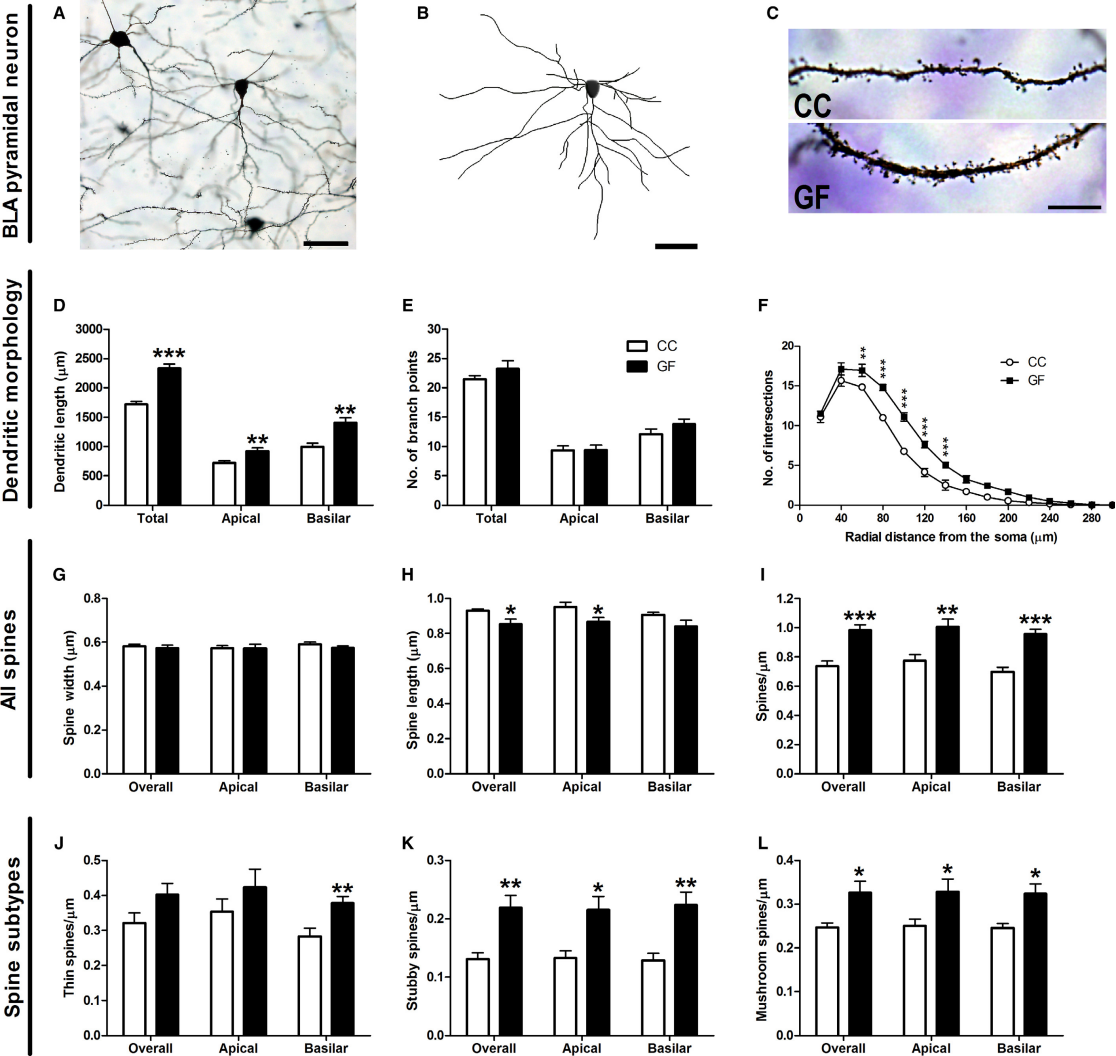
Dendritic elongation of BLA pyramidal neurons in GF mice. (A, B) Representative photomicrograph of a Golgi‐stained pyramidal neuron in the BLA (A) and resulting computer‐assisted morphometric reconstruction (B). Scale bars = 50 μm. (C) Representative photomicrographs of BLA pyramidal neuron dendritic spine density in CC and GF mice. Scale bar = 10 μm. (D, E) The apical and basilar dendrites of BLA pyramidal neurons of GF mice were significantly longer (D), but showed no alterations in branching compared to CC animals (E). (F) In GF mice, Sholl analysis revealed an extension of proximal and intermediate dendrites (60–140 μm from the soma). (G) There was no change in the width of dendritic spines between groups. (H) Spines were shorter in GF mice. (I) GF mice had more spines over the entire dendritic arbour of BLA pyramidal neurons compared to CC controls. (J, K, L) This proliferation of spines was accounted by an increase in thin spines (J), stubby spines (K) and mushroom spines (L). CC,* n* = 6; GF,* n* = 7. *P ≤* *0.05, **P <* *0.01, ***P <* *0.001.

The dendrites of pyramidal neurons were significantly longer in GF compared to CC mice (Fig. 3). In GF animals, total dendritic length was increased by 36% (*t*_11_ = 6.28, *P *<* *0.001; Fig. 3D); however, GF status had no effect on the total number of branch points (*t*_11_ = 1.09, *P* = 0.30; Fig. 3E). Similarly, in GF mice apical and basilar dendritic lengths were significantly elongated by 28 and 41%, respectively, (apical: *t*_11_ = 3.15, *P* = 0.0093; basilar: *t*_11_ = 3.85, *P* = 0.0027; Fig. 3D) and there was no difference in apical or basilar branching between groups (apical: *t*_11_ = 0.038, *P* = 0.97; basilar: *t*_11_ = 1.37, *P* = 0.20; Fig. 3E). Two‐dimension Sholl analysis revealed a significant interaction of group (CC vs. GF) and distance from the soma on pyramidal neuron dendritic distribution (*F*_14, 154_ = 7.50, *P *<* *0.001). Subsequent *post hoc* analyses indicated that dendritic extension (14–100%; *P *<* *0.05) was localized to the proximal and intermediate portions (60–140 μm) of the arbour (Fig. 3F).

The spine density of each BLA pyramidal neuron was characterized by sampling apical and basilar dendritic segments (Fig. 3G–L). The data set comprised a total of ~ 7700 dendritic spines from 78 neurons. The width and length of each individual spine was measured. Spines were then classified into thin, mushroom, stubby and filopodia subtypes using these measurements. There was no difference in spine width between GF and CC mice on any portion of the dendritic arbour (overall: *t*_11_ = 0.49, *P* = 0.63; apical: *t*_11_ = 0.050, *P* = 0.96; basilar: *t*_11_ = 1.31, *P* = 0.21; Fig. 3G). The length of spines was 8% shorter overall and 9% shorter on apical dendrites; however, there was no difference in the length of spines on basilar dendrites (overall: *t*_11_ = 2.34, *P* = 0.039; apical: *t*_11_ = 2.35, *P* = 0.038; basilar: *t*_11_ = 1.65, *P* = 0.13; Fig. 3H). The overall spine density (includes all spine subtypes) was increased by 33% in GF mice (*t*_11_ = 4.85, *P *<* *0.001; Fig. 3I). This increase in spines was consistent over the entire dendritic arbour: spine density was increased by 29 and 38% on apical and basilar dendrites, respectively (apical: *t*_11_ = 3.48, *P* = 0.0052; basilar: *t*_11_ = 5.68, *P *<* *0.001; Fig. 3I). There were 34% more thin spines on the basilar dendrites of GF vs. CC mice, but no significant change in thin spine density overall and on apical dendrites (overall: *t*_11_ = 1.85, *P* = 0.092; apical: *t*_11_ = 1.09, *P* = 0.30; basilar: *t*_11_ = 3.32, *P* = 0.0069; Fig. 3J). The overall stubby spine density was increased by 67% in GF mice compared to controls (overall: *t*_11_ = 3.43, *P* = 0.0056; Fig. 3K). This increase in stubby spines was observed in all portions of the dendritic arbour: there were 62% and 73% more stubby spines on the apical and basilar dendrites of GF mice, respectively (apical: *t*_11_ = 3.08, *P* = 0.011; basilar: *t*_11_ = 3.49, *P* = 0.0050; Fig. 3K). In GF mice, mushroom spines were similarly increased by 32% overall, by 31% on apical dendrites and by 32% on basilar dendrites (overall: *t*_11_ = 2.69, *P* = 0.021; apical: *t*_11_ = 2.23, *P* = 0.047; basilar: *t*_11_ = 2.96, *P* = 0.013; Fig. 3L). There was no change in filopodia spine density on any portion of the dendritic arbour in CC vs. GF mice (overall: *t*_11_ = 0.66, *P* = 0.52; apical: *t*_11_ = 0.23, *P* = 0.82; basilar: *t*_11_ = 0.96, *P* = 0.36; data not shown). Filopodia spine data are not shown as this subtype represents less than 5% of all spines.

#### Ventral hippocampal pyramidal neuron dendritic morphology and spine density

**Figure 4:**
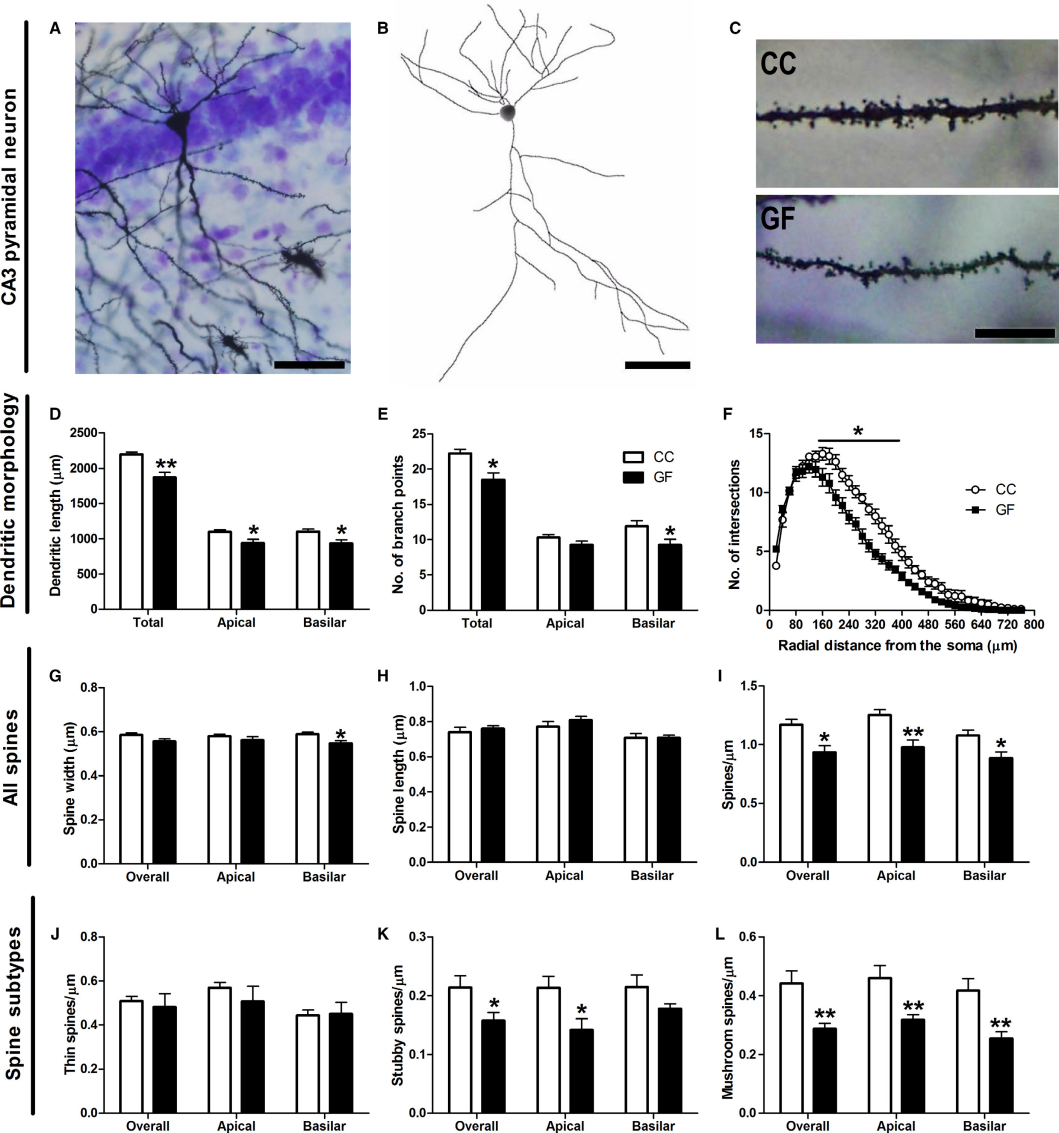
Dendritic atrophy of ventral hippocampal pyramidal neurons in GF mice. (A, B) Representative photomicrograph of a Golgi‐stained pyramidal neuron in the ventral hippocampus (A) and resulting computer‐assisted morphometric reconstruction (B). Scale bars = 50 μm. (C) Representative photomicrographs of ventral hippocampal pyramidal neuron dendritic spine density in CC and GF mice. Scale bar = 10 μm. (D, E) The dendrites of ventral hippocampal pyramidal neurons of GF mice were significantly shorter (D) and had less branching compared to controls (E). (F) In GF mice, Sholl analysis revealed a retraction in intermediate dendrites (160–380 μm from the soma). (G, H) The spines on basilar dendrites were thinner in GF mice (G), but there was no significant difference in the length of spines between groups (H). (I) GF mice had less spines on all portions of the dendritic arbour of ventral hippocampal pyramidal neurons compared to CC animals. (J, K, L) This reduction in spine density was due to a decrease in stubby spines (K) and mushroom spines (L), but not of thin spines (J). CC,* n* = 5; GF,* n* = 6. *P ≤* *0.05, **P <* *0.01.

When compared to CC controls, the ventral hippocampal pyramidal neurons of GF mice were significantly shorter and less complex (Fig. 4D–F). In GF animals, the total, apical and basilar dendritic lengths were all reduced by 15% (total: *t*_9_ = 3.72, *P* = 0.0048; apical: *t*_9_ = 2.43, *P* = 0.038; basilar: *t*_9_ = 2.59, *P* = 0.029; Fig. 4D). The total number of branch points was similarly decreased by 17% in GF mice (*t*_9_ = 3.07, *P* = 0.013; Fig. 4E). This reduction in branching was localized to the basilar dendritic arbour: the basilar dendrites of GF mice had 22% less branch points compared to controls, but apical dendritic branching did not differ between groups (apical: *t*_9_ = 1.47, *P* = 0.18; basilar: *t*_9_ = 2.59, *P* = 0.029; Fig. 4E). Two‐dimension Sholl analysis revealed a significant interaction of group (CC vs. GF) and distance from the soma on pyramidal neuron dendritic distribution (*F*_37, 333_ = 5.99, *P *<* *0.001); *post hoc* comparisons specified that the loss of dendritic material (15–41%; *P *<* *0.05) occurred in the intermediate portions (160–380 μm) of the arbour (Fig. 4F).

Spine morphology and density of each ventral hippocampal pyramidal neuron was measured by sampling apical and basilar dendritic segments (Fig. 4G–L). The data set comprised a total of ~ 7900 dendritic spines from 66 neurons. When compared to CC mice, there was no difference in spine width overall or in the apical dendritic arbour of GF mice; however, the width of basilar dendritic spines was 7% smaller in GF mice (overall: *t*_9_ = 1.86, *P* = 0.096; apical: *t*_9_ = 0.99, *P* = 0.35; basilar: *t*_9_ = 2.68, *P* = 0.025; Fig. 4G). There was no difference in the length of spines overall or on apical and basilar dendrites between groups (overall: *t*_9_ = 0.67, *P* = 0.52; apical: *t*_9_ = 0.99, *P* = 0.35; basilar: *t*_9_ = 0.019, *P* = 0.98; Fig. 4H). The overall spine density (includes all spine subtypes) was 20% lower in GF mice (*t*_9_ = 3.20, *P* = 0.011; Fig. 4I). This reduction in spines occurred in both the apical and basilar portions of the dendritic arbour: spine density was reduced by 22% on apical dendrites and by 18% on basilar dendrites (apical: *t*_9_ = 3.48, *P* = 0.0070; basilar: *t*_9_ = 2.76, *P* = 0.022; Fig. 4I). There was no significant difference in thin spine density in GF vs. CC mice (overall: *t*_9_ = 0.39, *P* = 0.71; apical: *t*_9_ = 0.79, *P* = 0.45; basilar: *t*_9_ = 0.11, *P* = 0.91; Fig. 4J). The overall stubby spine density was significantly reduced in GF animals by 26% (*t*_9_ = 2.42, *P* = 0.039; Fig. 4K). This loss in stubby spines was localized to the apical portion of the dendritic tree: stubby spine density was 33% lower on apical dendrites, but there was no such loss of stubby spines on basilar dendrites (apical: *t*_9_ = 2.63, *P* = 0.028; basilar: *t*_9_ = 1.81, *P* = 0.10; Fig. 4K). When compared to controls, the overall mushroom spine density was reduced by 35% in GF mice (*t*_9_ = 3.57, *P* = 0.0065; Fig. 4L). This loss of mushroom spines was distributed throughout the dendritic arbour: mushroom spine density was reduced by 31% on apical dendrites and by 39% on basilar dendrites (apical: *t*_9_ = 3.32, *P* = 0.0090; basilar: *t*_9_ = 3.57, *P* = 0.0060; Fig. 4L). Finally, there was no difference in filopodia spines between groups (overall: *t*_9_ = 0.70, *P* = 0.50; apical: *t*_9_ = 0.58, *P* = 0.58; basilar: *t*_9_ = 0.66, *P* = 0.52; data not shown).

#### Dorsal hippocampal dentate granule cell dendritic morphology and spine density

**Figure 5:**
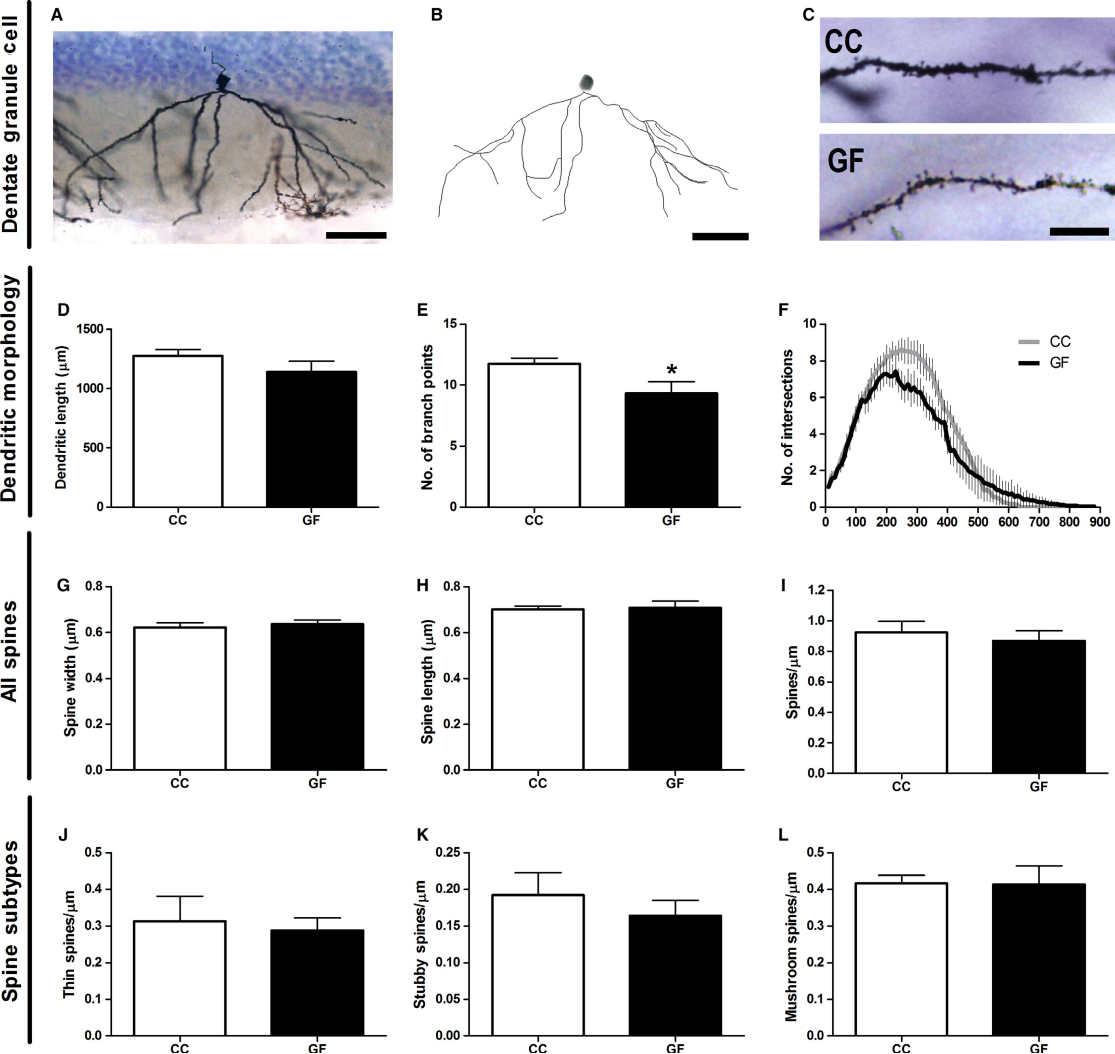
Reduced branching of dentate granule cells in GF mice. (A, B) Representative photomicrograph of a Golgi‐stained dentate granule cell in the dorsal hippocampus (A) and resulting computer‐assisted morphometric reconstruction (B). Scale bars = 50 μm. (C) Representative photomicrographs of granule cell spine density in CC and GF mice. Scale bar = 10 μm. (D, E) The dendrites of granule cell pyramidal neurons of GF mice did not differ in length (D), but had less branching compared to controls (E). (F) In GF mice, Sholl analysis revealed a loss of dendritic complexity; however, *post hoc* comparisons revealed no statistically significant distances in which this reduction in branching occurred. (I, J, K, L) There was no difference in the density of all (I), thin (J), stubby (K) or mushroom (L) spines in GF versus CC mice. *n* = 6 for both groups. *P ≤* *0.05.

The dorsal dentate granule cells of GF mice were less complex compared to controls (Fig. 5A–F). In GF mice, there was no change in granule cell dendritic length between groups (*t*_10_ = 1.29, *P* = 0.23; Fig 5D); however, there was a 21% reduction in the number of branch points (*t*_10_ = 2.29, *P* = 0.045; Fig 5E). Two‐dimension Sholl analysis revealed a significant interaction of group (CC vs. GF) and distance from the soma on the distribution of granule cell dendrites (*F*_87, 870_ = 1.48, *P* = 0.0042); however, *post hoc* comparisons uncovered no statistically significant dendritic regions in which the reduction in complexity occurred (Fig. 4F).

Spine morphology and density of each dorsal hippocampal dentate granule cell was measured by sampling dendritic segments throughout the arbour (Fig. 5G–L). The data set included a total of ~ 2600 dendritic spines from 48 neurons. There was no difference in spine width or length between GF and CC mice (width: *t*_10_ = 0.49, *P* = 0.63; length: *t*_10_ = 0.21, *P* = 0.83; Fig. 5G–H). Granule cell spine density also did not differ between groups (*t*_10_ = 0.57, *P* = 0.57; Fig. 5I). Spine subtype analysis revealed no differences in the densities of thin, stubby, mushroom or filopodia spines in CC vs. GF mice (thin: *t*_10_ = 0.33, *P* = 0.75; stubby: *t*_10_ = 0.75, *P* = 0.47; mushroom: *t*_10_ = 0.062, *P* = 0.95; filopodia: *t*_10_ = 0.83, *P* = 0.42 [data not shown]; Fig. 5J‐L).

## Discussion

This study explored how the microbiota affects the morphology of the amygdala and hippocampus, key brain regions implicated in regulating the range of behavioural and physiological endpoints which are abnormal in GF animals. Our data show, for what is to our knowledge the first time, that mice lacking a gut microbiota displayed an enlarged amygdala and hippocampus. Importantly, there was no difference in total brain volume between GF and CC mice, ruling out the possibility that these enlargements were due to whole‐brain expansion. Germ‐free status induced dendritic hypertrophy in BLA inhibitory aspiny interneurons and excitatory pyramidal neurons. BLA pyramidal neurons also showed increased thin, stubby and mushroom spine density. When the increase in dendritic length and spine density is taken together, we estimate that there are 81% more axospinous synapses on the BLA pyramidal neurons of GF mice. In contrast, the absence of a gut microbiota induced dendritic atrophy in both hippocampal pyramidal neurons and dentate granule cells. In GF mice, there was also a loss of stubby and mushroom spines on hippocampal pyramidal neurons. We estimate that there are 32% fewer synaptic connections on the hippocampal pyramidal neurons of GF mice when the decreases in dendritic length and spines are combined. Taken together, our findings demonstrate that the gut microbiota is necessary for the normal morphology of the amygdala and hippocampus and suggest that structural changes could underlie the altered stress responses and behavioural profile observed in GF mice.

### Morphological impact of growing up germ free

Increasing evidence from GF mice points to a role for the microbiota in regulating social and stress‐related behaviours relevant to emotional and neurodevelopmental disorders such as anxiety, depression and autism spectrum disorder (Sudo *et al*., 2004; Heijtz *et al*., 2011; Neufeld *et al*., 2011; Clarke *et al*., 2013; Desbonnet *et al*., 2014; Luczynski *et al*., 2016). However, the neurobiological mechanisms underlying these phenotypes are unclear. To this end, we compared the striking morphological changes observed in GF mice to those found in animal models of stress‐related disorders and autism.

#### Amygdalar and hippocampal volumes

Disruption of social behaviour, a characteristic of both animal models of autism and the GF mouse (Desbonnet *et al*., 2014; Arentsen *et al*., 2015), is associated with a alterations in amygdala and hippocampus volume. The effect of prenatal valproate exposure, a risk factor for autism spectrum disorder, on the structural development of the amygdala remains unclear: both reduced (Sosa‐Díaz *et al*., 2014) and increased (Olde Loohuis *et al*., 2015) BLA area sizes have been reported. In the hippocampus, valproate exposure increases hippocampal cell density (Edalatmanesh *et al*., 2013), whereas maternal immune activation, another model of autism in animals, reduces amygdalar volume and hippocampal thickness (Fatemi *et al*., 1999, 2008; Abazyan *et al*., 2010).

Changes in amygdalar and hippocampal size have also been documented in rodents subjected to stressors. Prenatally stressed rats have increased LA volume (Salm *et al*., 2004) and both chronic stress and corticosterone treatment induce hippocampal atrophy (Isgor *et al*., 2004; Murray *et al*., 2008; Lee *et al*., 2009). It remains unclear how gross changes in amygdalar and hippocampal volume relate to stress‐related brain function. However, the fact that changes in the size of these structures are frequently observed in humans with anxiety disorders or autism clearly indicates that volumetric alterations of limbic structures either influence or are a result of maladaptive stress responsivity (De Bellis *et al*., 2000; Schumann *et al*., 2004, 2011; Salzer & Weniger, 2010; Machado‐de‐Sousa *et al*., 2014). To further explore what the changes in amygdalar and hippocampal volume could mean for the stress‐related brain function of GF mice, we analysed the dendritic morphology and synaptic connectivity of single neurons in these regions (see below).

#### Amygdalar and hippocampal dendritic morphology

We found dendritic hypertrophy of BLA aspiny interneurons in GF mice. Dendritic hypertrophy of GABAergic inhibitory interneurons has also been observed in the prefrontal cortex of chronically stressed rodents (Gilabert‐Juan *et al*., 2013). However, further investigation is required to determine if dendritic remodelling of interneurons is limited to the BLA and if it is induced by other treatments.

There exists a substantial literature documenting morphological changes to excitatory pyramidal neurons in the BLA of rodents with similar behavioural profiles to GF mice. In the autism mouse model induced by exposure to valproate, BLA neurons are longer but have reduced spine densities (Bringas *et al*., 2013). Chronic stress induces a long‐lasting hypertrophy and increased spine density of rat BLA pyramidal neurons (Vyas *et al*., 2002, 2004, 2006).

Dendritic remodelling in hippocampal neurons has been observed in rodent models which, like GF mice, display altered stress responsivity and sociability. Most notably, repeated stress induces apical dendritic atrophy and spine loss in CA3 pyramidal neurons (Watanabe *et al*., 1992; Magariños & McEwen, 1995b; Vyas *et al*., 2002; Sandi *et al*., 2003; Christian *et al*., 2011). Interestingly, repeated stress and high fat diet act synergistically to induce dendritic atrophy in CA3 pyramidal neurons, raising the possibility that diet, metabolism and perhaps gastrointestinal function can impact brain structure (Baran *et al*., 2005). Although CA3 neurons have not yet been investigated in rodent models of autism, the CA1 neurons of these animals exhibit structural changes: maternal immune activation reduces dendritic length (Baharnoori *et al*., 2009), whereas prenatal exposure to valproate induces spine loss (Takuma *et al*., 2014).

Neurogenesis has a functional role in memory formation and stress responsivity (Snyder *et al*., 2011; Marín‐Burgin & Schinder, 2012), and is influenced by the microbiota (Ogbonnaya *et al*., 2015). A substantial portion of newly generated granule cells migrate into the granule layer of the dentate gyrus and become integrated into the hippocampal network (Hastings & Gould, 1999; Cameron & Mckay, 2001). We thus investigated if the morphology of mature dentate granule cells is also different in these animals, uncovering reduced branching in GF mice. Dentate granule cells appear to be resistant to structural remodelling induced by repeated corticosterone treatment (Woolley *et al*., 1990); however, maternal immune activation reduces dendritic complexity (Zhang & van Praag, 2015).

### Functional implications

#### HPA‐axis signalling

The amygdala has many downstream targets that modulate neuroendocrine stress responses (Herman *et al*., 2003; Ulrich‐Lai & Herman, 2009). The BLA is activated by psychological stressors and BLA lesions dampen HPA‐axis response to restraint (Cullinan *et al*., 1995; Bhatnager *et al*., 2004). The CeA, however, is not thought to be involved in HPA‐axis signalling to psychological stressors, but instead regulates autonomic stress responsivity (Dayas *et al*., 2001). In contrast to the amygdala, numerous studies have implicated the hippocampus with the inhibition of the HPA axis (Dunn & Orr, 1984; Sapolsky *et al*., 1984; Herman *et al*., 2005). GF mice show increased HPA‐axis signalling in response to acute restraint stress (Sudo *et al*., 2004; Neufeld *et al*., 2011; Clarke *et al*., 2013). Thus, the increase in HPA‐axis signalling in GF mice could be due to concomitant BLA hyperactivation and hippocampal hypoactivation. Indeed, we found that the BLA of these animals is larger and neurons have increased spine density, suggesting that these cells receive more excitatory inputs. In the hippocampus, we found atrophy of both dentate granule cells and CA3 pyramidal cells in GF mice, implying that these cells receive less excitatory inputs. Taken together, we uncovered contrasting patterns of dendritic morphology in the amygdala and hippocampus of GF mice, which has marked implications for the neural regulation of HPA‐axis signalling.

#### Anxiety‐like and social behaviours

GF mice have reduced anxiety and deficits in social cognition (Heijtz *et al*., 2011; Neufeld *et al*., 2011; Desbonnet *et al*., 2014). There appears to be a significant link between anxiety and social behaviours. Recent studies have utilized optogenetics to confirm the involvement of both the amygdala and the ventral hippocampus in the regulation of these behaviours (Allsop *et al*., 2014). In addition to its role in anxiety (Deacon *et al*., 2002; Kjelstrup *et al*., 2002; Bannerman *et al*., 2004; McHugh *et al*., 2004), the ventral hippocampus is involved in sociability, with hippocampal lesions leading to abnormal responses engagement in social situations (Cadogan *et al*., 1994; Machado & Bachevalier, 2006; Kheirbek *et al*., 2013). Moreover, the ventral hippocampus has robust reciprocal connections with the amygdala, another brain structure involved in anxiety and sociability (Allsop *et al*., 2014).

The amygdalar subregions have distinct roles in the regulation of anxiety and social behaviours: the LA and BLA integrate sensory and aversive information and send projections to the CeA (Fanselow & LeDoux, 1999; Sah *et al*., 2003; Ledoux, 2007; Pape & Pare, 2010; Allsop *et al*., 2014). In mice, stimulation of the projections from BLA to the CeA produced an anxiolytic phenotype (Tye *et al*., 2011). Importantly, stimulation of the BLA as a whole produced the opposite effect (Tye *et al*., 2011), suggesting that the majority of BLA neurons project to other sites and have an anxiogenic effect. Indeed, stimulation of BLA projections to the ventral hippocampus induced a state of increased anxiety but a reduction in sociability (Felix‐Ortiz *et al*., 2013; Felix‐Ortiz & Tye, 2014).

In this study, we report dendritic hypertrophy in the BLA and atrophy in the ventral hippocampus of GF mice. It is noteworthy that mushroom spine density was drastically impacted by microbial status: GF mice had one third more and less mushroom spines on BLA and ventral hippocampus pyramidal neurons, respectively. Mushroom spines are crucial for the formation of mature, long‐lasting glutamatergic synapses (Matsuzaki *et al*., 2001, 2004; Kasai *et al*., 2003; Holtmaat *et al*., 2005); therefore, changes in their number likely have a significant influence on the number of excitatory inputs the neurons of GF mice receive. In keeping with this idea, our data suggest that the BLA is hyperactive, whereas the ventral hippocampus is hypoactive. Indeed, transcriptomic analysis revealed that the amygdala of GF mice is in a hyperactive state (Stilling *et al*., 2015). However, the GF behavioural profile – reduced anxiety and social cognition – clearly cannot be explained by either excitation or inhibition of the BLA–ventral hippocampal circuit alone but instead may reflect the cumulative impact of relevant pathways.

The functional relationship between amygdalar and hippocampal activity and social and anxiety behaviours is likely more complex; the BLA and ventral hippocampus project and receive inputs from many other brain areas involved in these behaviours including sensory regions such as sensory thalamus and sensory cortices, the prefrontal cortex, the lateral septum, the supraoptic nucleus, the paraventricular nucleus, the periaqueductal grey and the bed nucleus of the stria terminalis (Herman *et al*., 2003; Ledoux, 2007; Ulrich‐Lai & Herman, 2009; Janak & Tye, 2015). Interestingly, GF mice show hypermyelination of the prefrontal cortex (Hoban *et al*., 2016), further enforcing the idea that neural communication is altered in several key brain structures.

### Future directions

This study serves as a proof of principle experiment which raises the intriguing possibility that the gut microbiota is required for normal brain structure. However, the developmental time point during which the microbiota exerts its influence remains to be determined. The structural brain changes observed may not be the result of a defective brain–gut axis during postnatal life, but rather could be due to the absence of microbiota in the dam during embryogenesis and gestation. Indeed, the peripheral hormones of the mother, which could also include gut hormones, are known to play key organizational roles in the brain during gestation (Bale *et al*., 2010; Buss *et al*., 2012). Moreover, prenatal stress has been shown to affect the microbiota in both rodents and humans (Golubeva *et al*., 2015; Jašarević *et al*., 2015; Zijlmans *et al*., 2015). Recent evidence has demonstrated that the maternal microbiota during pregnancy also impacts the immune function of the offspring (Gomez de Agüero *et al*., 2016). Alternatively, the observed changes in brain structure in GF mice could be occurring during the postnatal period. To tease apart the pre‐ versus postnatal influence of the microbiota on brain development, the amygdala and hippocampus structure of GF and CC mice should be investigated at several time points. To eliminate the possibility that the microbiota could influence brain development prenatally, future studies should also deplete the microbiota with antibiotics at different postnatal time points. These experiments would allow researchers to determine if the alterations in dendritic spines in the amygdala and hippocampus occur because of deficits in the initial synaptogenesis or later pruning of spines during development (Tau & Peterson, 2009).

An intriguing question that arises from this study is whether colonizing GF mice with bacteria could prevent or reverse the observed structural brain changes. This is especially of interest given that chronic treatment with *Lactobacillus rhamnosus* reduces anxiety and alters GABA receptor expression in the amygdala (Bravo *et al*., 2011). Colonization experiments have been performed in GF mice, with certain behavioural and physiological abnormalities proving reversible (Sudo *et al*., 2004; Heijtz *et al*., 2011; Clarke *et al*., 2013; Desbonnet *et al*., 2014) and others irreversible (Clarke *et al*., 2013; Desbonnet *et al*., 2014; Ogbonnaya *et al*., 2015; Stilling *et al*., 2015). Moreover, there appear to be critical time windows in which HPA‐axis signalling can be normalized in GF mice (Sudo *et al*., 2004); therefore, the time point in which bacteria is introduced should be carefully considered (Borre *et al*., 2014; Neufeld *et al*., 2016).

## Conclusions

Our results show altered brain morphology in GF animals and suggest that the amygdala and hippocampus are brain regions whose structural integrity is contingent on the presence of a gut microbiota. This has implications for the expression of limbic system‐mediated behaviours and physiology relevant to the stress response, anxiety‐like behaviours and social interactions. Further studies are warranted to establish if amygdalar and hippocampal structure are malleable at a structural level following microbiota‐directed interventions across the lifespan. An increased understanding of the impact of the microbiota on these brain structures may ultimately inform novel strategies for the treatment of multiple neuropsychiatric disorders.
